# Immunization strategies to prevent malaria in pregnancy – a multistakeholder workshop

**DOI:** 10.1016/j.vaccine.2026.128505

**Published:** 2026-04-19

**Authors:** Flavia D'Alessio, Josiane Honkpehedji, Sodiomon Sirima, Benoit Gamain, Nielsen Morten, Nicaise Ndam, Benjamin Mordmüller, Alassane Dicko, Rhanda Adechina, Ange Dossou, Jobiba Chinkhumba, Mandeep Kaur, Roch Houngnihin, D. Scott LaMontagne, Clara Menendez, Adidja Amani, Tendai Mayani, Mary J. Hamel, Elvira Carrió, Aicha Sayeh, Ayola Akim Adegnika

**Affiliations:** a European Vaccine Initiative, EVI, Germany; b La Fondation pour la Recherche Scientifique, FORS, Benin; c Groupe de Recherche Action en Santé, GRAS, Burkina Faso; d Sorbonne Université, INSERM, CNRS, Centre d'Immunologie et des Maladies Infectieuses, CIMI F-75013 Paris, France; e University of Copenhagen, UCPH, Denmark; f Institut de Recherche pour le Développement, IRD, France; g Radboud University Medical Center, Radboudumc, the Netherlands; h Malaria Research and Training Center, University of Science, Techniques and Technologies of Bamako, Bamako, Mali; i African Vaccine Regulatory Forum (AVAREF), Brazzaville, Congo; j Ministry of Health, Benin; k Kamuzu University of Health Sciences, KUHeS, Malawi; l University d'Abomey, Benin; m Internatinal Division, JSI Research & Training Institute, Arlington, VA, USA; n Barcelona Institute for Global Health, ISGlobal, Spain; o AMVIRA (Accelerated Malaria Vaccine Introduction and Roll-out in Africa), WHO Regional Office for Africa, Brazzaville, Congo; p Mothers2Mothers, South Africa; q Independent Consultant, Switzerland; r P95 Clinical and Epidemiology Services, Leuven, Belgium; s Centre de Recherces Medicales de Lambarene, CERMEL, Gabon; t German Center for Infection Research (DZIF), Partner site Tübingen, Tübingen, Germany; u Institut für Tropenmedizin, Universitätsklinikum Tübingen, Germany

**Keywords:** Malaria in pregnancy, Placental malaria, Vaccine development, Vaccine implementation

## Abstract

As part of the ADVANCE-VAC4PM project, the European Vaccine Initiative (EVI) and La Fondation pour la Recherche Scientifique (FORS) co-hosted a hybrid workshop titled “Strategies for using malaria vaccines to prevent malaria in pregnancy (MiP)”. The event brought together researchers, regulators, civil society, global health experts, and policymakers to discuss the need for MiP vaccines and strategies for their development, evaluation, and implementation.

Malaria remains a major global health threat, with sub-Saharan Africa bearing the highest burden. Pregnant women (PW) are highly vulnerable, with an estimated 12.4 million affected in 2023. Beyond maternal health effects, a major cause of the MiP-related disease burden is placental malaria (PM), which can cause significant morbidity in newborns. PM risk is greatest in primigravidae and secundigravidae, as immunity develops over successive pregnancies. As existing malaria control strategies remain insufficient, MiP vaccines have the potential to complement them by eliciting immunity comparable to that seen in multigravidae. To be effective, such a vaccine should provide long-lasting immunity and target adolescent girls and women before their first pregnancy. PM vaccine candidates targeting the VAR2CSA antigen (PRIMVAC and PAMVAC) are in development, and existing malaria vaccines preventing infection are being repurposed to prevent MiP. However, limited awareness of MiP-specific burden, weak pharmacovigilance systems, and vaccine hesitancy may hinder future vaccine implementation. Key recommendations highlighted during the workshop included strengthening communication and community engagement strategies, defining relevant efficacy endpoints for pivotal clinical trials, reinforcing pharmacovigilance systems to support safety and real-world effectiveness studies, and planning for early regulatory alignment. Panel discussions emphasized the importance of stakeholder coordination and reduced-dose schedules to support future MiP vaccine programmatic feasibility. The workshop concluded with a call for sustained collaboration and national investment to ensure that MiP vaccines become a viable and effective component of global malaria prevention efforts.

## Highlights

• Malaria in pregnancy (MiP) poses a high disease burden in sub-Saharan Africa.
• Vaccination, combined with current interventions, may reduce MiP.
• PM-specific and general malaria vaccines are in clinical development to prevent MiP.
• Communication and community engagement are key for MiP vaccine acceptability.
• Early regulatory engagement is needed for MiP vaccine development.

## 1 Introduction

Malaria remains a major global health concern, with sub-Saharan Africa carrying the greatest burden. Pregnant women (PW) are especially vulnerable to malaria in pregnancy (MiP), even in areas with stable transmission and prior partial immunity to *Plasmodium falciparum*. Moreover, this elevated risk may persist into the postpartum period [1]. MiP is characterized by parasitaemia during pregnancy, which can lead to placental malaria (PM). PM is defined by the accumulation of maternal infected red blood cells (iRBCs) in the placenta. Here, maternal infected erythrocytes can bind to chondroitin sulfate A (CSA) with the parasite-encoded surface protein variant surface antigen 2 – CSA (VAR2CSA), a *P.falciparum e*rythrocyte membrane protein 1 (PfEMP1) family protein [2]. This interaction causes inflammation and placental dysfunction, associated with poor birth outcomes, including low birth weight and stillbirths. Primi- and secundigravidae are at higher risk of PM than multigravidae [3], [4], as immunity builds over successive pregnancies exposed to malaria through IgG antibodies that block VAR2CSA binding [5].

Current MiP prevention strategies include intermittent preventive treatment in pregnancy (IPTp) with sulfadoxine-pyrimethamine (SP), insecticide-treated bed nets (ITNs), and prompt diagnosis and treatment of clinical malaria. Nevertheless, a significant proportion of PW at risk is still not benefiting from these protective interventions. IPTp-SP is only initiated at 13 weeks of gestation, although increasing evidence suggests that *P. falciparum* infection is particularly frequent and detrimental to the fetus in the first trimester of pregnancy [6], [7]. In this context, complementary prophylactic measures such as vaccination might contribute to reducing MiP burden.

Two VAR2CSA-derived vaccines are being developed for use in nulligravid girls, aiming to mimic naturally acquired immunity to PM: PAMVAC [8] and PRIMVAC [9]. To promote the clinical development of these vaccine candidates, the European Vaccine Initiative (EVI) launched the ADVANCE-VAC4PM project in 2022. As part of this initiative, a hybrid workshop titled “Strategies for using malaria vaccines to prevent malaria in pregnancy” was held on June 3, 2025 La Fondation pour la Recherche Scientifique (FORS), in Cotonou (Benin), co-hosted by EVI and FORS. The event brought together around 200 participants (of which 60 attended in person at FORS), including researchers, regulators, civil society, global health experts, and policymakers to discuss the need for vaccines for MiP and strategies for their development, evaluation, and implementation. Lessons from IPTp and human papillomavirus (HPV) vaccination programs were also discussed to guide future efforts.

## 2 Overview of MiP in sub-Saharan Africa and current control strategies

Following the opening remarks by **Dr Flavia D'Alessio** (EVI, Germany) and **Dr Ange Dossou** (Ministry of Health (MoH) of Benin representative), **Dr. Sodiomon Sirima** (Groupe de Recherche Action en Santé, GRAS, Burkina Faso) presented an overview of the burden, pathogenesis, and current control strategies of MiP in sub-Saharan Africa.

In 2023, 36 million PW were at risk of MiP in the World Health Organization (WHO) African Region, and 34% (12.4 million) were infected. Prevalence was highest in West Africa (36.4%, 6 million PW) and Central Africa (40.5%, 3.4 million PW) [10]. MiP poses risks to both mothers and newborns. Infected mothers face increased risk of anemia (2.4-fold) [11], severe malaria (3-fold), including higher risk of renal failure, pulmonary oedema, and cerebral malaria [12]. For newborns, MiP raises the risk of preterm birth (1.6-fold), stillbirth (1.4-fold), and low birth weight (2-fold) [13], [14]. Maternal infection can also lead to congenital malaria, affecting up to 33% of exposed infants [15]. Risk factors for MiP include living in moderate or high transmission areas, primigravidity, young maternal age, timing of infection, human immunodeficiency virus (HIV) co-infection, and frequent malaria episodes [16], [17].

MiP case management relies on clinical evaluation and parasitological confirmation by microscopy or rapid tests. Antimalarial treatment is tailored to the specific trimester of pregnancy and disease severity [18]. Artemether-lumefantrine (AL) is given in the first trimester, and any artemisinin combined therapy (ACT, such as AL, dihydroartemisinin [DHA], artesunate-amodiaquine [AS-AQ], artesunate-mefloquine [AS-MQ], artesunate-pyronadine [ASPY]) is given in the second and third trimester. Severe malaria is treated with injectable artesunate [18].

The primary strategies to prevent MiP are based on the use of ITNs and IPTp-SP. ITN use is increasingly adopted, with a coverage of 60% in 2023 [10], but IPTp-SP coverage remains suboptimal (see section 5.7).

Beyond the limited coverage of current control strategies, these proven interventions are threatened by widespread resistance to standard insecticides and SP, as well as delayed parasite clearance following treatment with ACTs. In response, since 2023, the WHO has recommended two new types of ITNs [19]. For prevention during pregnancy, alternative drugs for IPTp are under evaluation, especially for HIV-infected women in whom SP is contraindicated [20]. In the context of recent WHO recommendations for malaria vaccines RTS,S/AS01 (2021) and R21/Matrix-M (2023) for pediatric use [21], the potential of a MiP vaccine is being pursued.

## 3 Vaccine candidates against MiP: Status of research & development

**Dr. Benoit Gamain** (Institut National de la Santé et de la Recherche Médicale, INSERM, France) presented the status of the PRIMVAC vaccine candidate and the ADVANCE-VAC4PM project. PRIMVAC expressed in *Escherichia coli* SHuffle® is based on the DBL1x-2x region of the VAR2CSA (3D7 strain) and was selected as the optimal region for developing a PM vaccine [22], [23]. In 2020, the PRIMALVAC Phase Ia/Ib trial demonstrated that three doses of PRIMVAC, adjuvanted with Alhydrogel or glucopyranosyl lipid adjuvant-stable emulsion (GLA-SE), were safe and well tolerated, achieving 100% seroconversion, with 93% remaining seropositive after one year [9]. Following this success, the GHIT-funded VAC4PM project was launched in October 2021 to study long-term immunity and the boosting effects of PRIMVAC. Preliminary findings indicate that over 80% of vaccinated PW retain antibodies seven years after vaccination (NCT05426187). As part of VAC4PM, a Phase Ib trial is under preparation in Burkina Faso to administer a single dose of PRIMVAC to nulligravid, primigravid, and multigravid women and evaluate its ability to boost naturally acquired VAR2CSA immunity.

Building on these efforts, the EU-funded ADVANCE-VAC4PM project was launched in June 2022 and will run until May 2027. The project seeks to improve and broaden the immune response induced by PRIMVAC and PAMVAC (described by Dr. Nielsen) by using a novel delivery platform for PAMVAC based on capsid virus-like particles (cVLPs) and evaluating co-administration of PRIMVAC and PAMVAC-cVLP.

In parallel, a PRIMVAC Phase II trial in African nulligravid women is in preparation, and an experimental mRNA-based PRIMVAC vaccine candidate is being developed.

**Dr Morten A. Nielsen** (University of Copenhagen, UCPH, Denmark) provided an overview of the development progress of the PAMVAC-cVLP vaccine candidate. This vaccine is a re-engineered version of the PAMVAC antigen candidate spanning the CSA-binding ID1-ID2a region of the FCR3- previously produced in insect cells and now produced in *E. coli* SHuffle®. PAMVAC was previously tested in a Phase Ia/Ib clinical trial, demonstrating that the vaccine was safe, well tolerated, and induced a strong immune response in adult malaria-naive volunteers [8].

In the PAMVAC-cVLP vaccine, the PAMVAC antigen is coupled to cVLPs to focus the immune response specifically on the outermost regions of the antigen, critical for blocking parasite adhesion. Preclinical mouse studies showed that the cVLP strategy enhanced PAMVAC antibody responses and prolonged their duration. The same cVLP platform was tested in a Phase 1 SARS-CoV-2 vaccine trial, showing good tolerability and strong, long-lasting immune response after two doses [24]. In a booster setting, the non-adjuvanted vaccine was non-inferior to mRNA vaccines (Comirnaty™) and showed excellent safety (unpublished).

The PAMVAC-cVLP *E.coli-*based production model uses globally supplied materials, enabling manufacturing in low- and middle-income countries (LMICs) and facilitating future implementation. cGMP manufacturing started in June 2025, with a Phase Ia/Ib clinical trial planned for early 2026.

**Dr. Nicaise Ndam** (Institut de Recherche pour le Développement, IRD, France) presented a mosaic cVLP approach for the combination of VAR2CSA-based antigens derived from three different variants. Despite early clinical trials confirming that the PM vaccine candidates PAMVAC [8] and PRIMVAC [9] are safe and immunogenic, low cross-reactivity was observed due to VAR2CSA polymorphisms. To overcome this challenge, Dr. Ndam's team conducted a phylogenetic analysis of the ID1-DBL2X-ID2a region of VAR2CSA expressed by *P. falciparum* isolates from infected Beninese PW to identify antigenic diversity encountered in endemic regions. Three major clusters were defined [25].

To enhance the breadth and durability of the antibody response, the ID1-DBL2X-ID2a regions of a representative variant from each clade were attached to a cVLP scaffold. Preclinical studies confirmed that combining at least two to three variants enhanced vaccine performance, and mouse immunogenicity tests showed the induction of antibodies capable of cross-strain inhibition. The platform proved to be versatile, potentially allowing for co-delivery of antigens from other pathogens, such as HPV.

**Prof Alassane Dicko** (Malaria Research and Training Center, MRTC, Mali) presented recent efforts to conduct malaria vaccine trials in women of childbearing potential (WOCBP), aiming to extend protection into pregnancy (including the first trimester, see section 2.) by vaccinating before conception. This strategy has been applied in trials of the *Plasmodium falciparum* sporozoite (PfSPZ) and R21/Matrix-M vaccines.

The PfSPZ vaccine was evaluated in a double-blind, randomized controlled trial in Mali conducted by MRTC, National Institute of Allergy and Infectious Diseases, and Sanaria. The study involved 300 WOCBP (18–38 years) using contraception, randomized (1:1:1) to receive a low-dose, high-dose, or placebo. Those who became pregnant (57%) were followed through delivery. The vaccine was safe, well tolerated, and showed significant efficacy against *P. falciparum* parasitaemia and clinical malaria over two transmission seasons, also in PW [26].

Following WHO prequalification and recommendation for use in children living in moderate-to-high malaria transmission settings, the R21/Matrix-M malaria vaccine is now being evaluated in Mali in WOCBP aged 18–35 years in a clinical trial led by the University of Oxford, EVI, and MRTC (NCT06080243). Participants were randomized to receive two or three primary doses or a placebo, with a subset receiving a booster or placebo one year later. Contraception was maintained until 28 days post-final dose, and pregnancies were followed to assess vaccine safety and efficacy during gestation. Screening and enrollment were completed in May 2025, with initial results expected in 2026.

## 4 Clinical roadmap and regulatory framework for the MiP vaccine

**Prof. Benjamin Mordmüller** (Radboud University Medical Center, RUMC, The Netherlands) outlined critical considerations to guide progress toward PM vaccine licensure. Throughout the clinical development phases, key decision points need to be evaluated, including safety, tolerability, and immunogenicity in Phase I, the population in need, interaction with other interventions, efficacy, adjuvantation, and dosage in Phase II, and safety, efficacy, and effectiveness in Phases III and IV. Unique challenges for PM vaccines include the selection of appropriate clinical endpoints, such as fecundity (as a surrogate of prevention of miscarriage), placental infection, low birth weight, and maternal and neonatal mortality. Ultimately, licensure hinges on demonstrating that the benefits of vaccination outweigh the risks.

Systematic evaluation of vaccines in the context of existing interventions is recommended, along with the design of clinical trials that consider the biological and implementation-specific complexities of MiP. A major advantage of MiP vaccines over current control strategies (i.e. IPTp and ITN) is that they can be given before pregnancy to exert their protective effect already in early pregnancy. Corresponding study designs and endpoints to measure these effects need to be confirmed and standardized. As prevention of MiP will require multiple complementary interventions, even when MiP vaccines prove to be effective, it is particularly important to evaluate potential interactions within intervention to ensure that their effects on health outcomes are additive. Because PM vaccines may also be used to focus or boost immune responses to VAR2CSA (see presentation of Benoit Gamain, section 3), alternative clinical development scenarios shall be developed for that purpose.

In general, interventions to prevent MiP should not focus on pregnancy alone but include adolescence and the postpartum period. In the context of MiPvaccines, relevant efficacy endpoints for Phase II are still uncertain and should be explored and defined. Ongoing trials of PRIMVAC and PAMVAC will provide valuable insights into refining the developmental roadmap for the PM vaccines.

**Rhanda Adechina** presented the African Vaccine Regulatory Forum (AVAREF), a platform of African National Regulatory Authorities (NRAs) and ethics committees, focused on improving access to medical products across the continent.

AVAREF addresses regulatory gaps in Africa through four pillars: capacity building, expert support, partnerships and alliances for results, and a lifecycle approach to clinical trial development. Its initiatives strengthen national and regional regulation, support ethics committees, standardize procedures to improve efficiency, develop mentorship programs and offer multilingual training. AVAREF also supports the digitization of processes through an electronic clinical trial application platform for multi-country clinical trial submissions and coordinated reviews.

The platform serves as the Technical Committee of the clinical trial oversight function within the African Medicines Agency, supporting regulators while making Africa a more attractive and reliable environment for clinical research. It also provides scientific advice, joint review of clinical trials, and facilitates product registration. AVAREF's experience includes supporting the Phase III clinical trials and registration processes for malaria vaccines (RTS,S/AS01 (2021) and R21/Matrix-M), and it continues to offer its platform for evaluating new vaccine candidates, such as MiP vaccines.

## 5 MiP vaccine implementation in sub-Saharan Africa

### 5.1 WHO vaccine prequalification process

**Olivier Lapujade** (WHO) provided an overview of the WHO vaccine prequalification (PQ) process, a crucial mechanism that supports the international procurement of vaccines. PQ is not a regulatory authorization, but a WHO recommendation based on a rigorous assessment of quality, safety, efficacy, and programmatic suitability.

The PQ process relies on evaluations by NRA (for vaccine-producing countries only those benchmarked by the WHO as having at least maturity level 3 [27]), alignment with WHO standards and United Nations International Children's Emergency Fund (UNICEF) procurement requirements, consistent product characteristics, solid clinical data, and compliance with Good Manufacturing Practices. The standard PQ timeline is nine months (270 days internal time) but can be shortened to three when leveraging assessments from WHO-listed authorities. A critical element is the Programmatic Suitability for Prequalification (PSPQ), which ensures vaccines are practical for use in diverse settings, requiring thermostability (usually ≥ −20 °C), vial monitors, and suitable formats. Dossier submission for PQ requires: 1) registration by an NRA, 2) inclusion in the priority list [28], and 3) compliance with WHO recommendations. Post-PQ monitoring is also part of the process. With over 150 vaccines currently prequalified, WHO reviews safety and production reports, manages product variations, and investigates field-level complaints, especially on cold chain integrity.

Additionally, the Emergency Use Listing pathway allows time-limited recommendations for vaccines needed in public health emergencies, based on limited but essential data, with rigorous post-deployment monitoring and effectiveness studies.

### 5.2 Strategies for vaccine delivery to women of childbearing potential

**Dr Laura Nic Lochlainn** (WHO) presented WHO strategies for vaccinating women of childbearing age within a broader life course approach to immunization. Currently, WHO-recommended vaccines for PW include tetanus, diptheria and pertussis (TDaP) seasonal influenza, COVID-19 and respiratory syncytial virus. Vaccines in development target Group B Streptococcus, Ebola, and Zika. For adolescents, WHO WHO-recommended vaccines include boosters for tetanus, diphtheria, influenza, and HPV, with candidates for tuberculosis, gonococcus, and chikungunya under development [29].

The Maternal and Neonatal Tetanus Elimination (MNTE) initiative and HPV vaccine roll-out offer key lessons for future MiP immunization efforts, as they target similar populations. Launched in 1999, MNTE aims to vaccinate at least 80% of PW and women of reproductive age with tetanus-containing vaccine (TCV) in high-risk areas, promote clean deliveries, and strengthen surveillance. Ten countries have yet to eliminate tetanus, and hence, a supplementary strategy has been implemented: administering three TCV doses in high-risk zones. To sustain MNTE gains, the WHO recommends a life course approach: three infant TCV doses, one booster TCV dose in the second year of life, a second between 4 and 7 years of age, and a third between 9 and 15 years of age. Ideally, there should be at least four years between TCV booster doses, and six doses of TCV will confer long-term protection. However, global adoption varies-only 83 countries offer all three TCV boosters, while 43 offer none. Currently, Gavi, the Vaccine Alliance, supports only the second-year TCV booster; the rest require national funding.

HPV vaccine programs show the value of school-based delivery, though implementation varies. Globally, about 126 countries report school vaccination checks, despite these platforms remain underused in many LMICs. The need to vaccinate”hard-to-reach” and underserved populations was highlighted.

### 5.3 Introduction of the MiP vaccine into the Expanded Programme on Immunization

**Dr. Ange Dossou's** (MoH, Benin) intervention focused on the Expanded Programme on Immunization (EPI) in sub-Saharan Africa, aimed to ensure access to essential vaccines. Launched by WHO in 1974, EPI is widely implemented and accepted across many African countries, contributing to a marked reduction in mortality and morbidity from diseases such as diphtheria, tetanus, polio, whooping cough, tuberculosis, and measles. Over the years, EPI has expanded to include vaccines against yellow fever, pneumococcus, rotavirus, rubella, and HPV for adolescent girls. The latest addition is the malaria vaccine, introduced in Benin on April 25, 2024.

For the successful introduction of the MiP vaccine, it is essential to prove efficacy, secure political commitment, and overcome financial, logistical, and community acceptance challenges. African countries and partners need to invest in local vaccine development and production to foster autonomy and public trust. Finally, regional collaboration was highlighted as key to sustainable immunization programs.

### 5.4 Cost-effective aspects to support MiP vaccine implementation

**Dr. Jobiba Chinkhumba** (Kamuzu University of Health Sciences, KUHeS, Malawi) shared preliminary results of the projected health and economic impacts of a PM vaccine targeting WOCBP. Using a decision tree model from a provider perspective, the analysis focused on a single pregnancy in a hypothetical cohort of 1000 women. The main outcome was neonatal life years gained by preventing stillbirths. Scenarios varied from best-case (high vaccine efficacy, low cost, high uptake) to worst-case (lower efficacy, higher cost, lower uptake). Results showed that targeting primigravida women is more likely to be cost-effective, with a baseline incremental cost-effectiveness ratio (ICER) of $273 per life year gained for neonates, which is well below the regional threshold of $776 (approximately half the gross domestic product per capita in sub-Saharan Africa). This remained favorable even under several less optimistic assumptions. In contrast, vaccinating multigravida women was less cost-effective due to lower risk to this population and their neonates, with a baseline ICER of $1405 and a probability of cost-effectiveness dropping significantly unless the willingness-to-pay threshold increases. A drop in vaccine efficacy as low as 5%, raised the ICER to $1800 for primigravidae and $9000 for multigravidae. A 50% cost increase raised the ICER to $400 and $2000, respectively. The analysis supported targeted vaccination strategies, particularly for primigravidae, to optimize resource use.

### 5.5 Socio-ethnographic aspects to support MiP vaccine implementation

Two socio-ethnographic studies were presented providing insights for future MiP vaccine acceptance.

**Mandeep Kaur** (EVI, Germany) presented findings from the ADVANCE-VACPM study in Malawi using interviews and focus groups with WOCBP, PW, family members, traditional birth attendants, counselors, and local leaders. Three main barriers to MiP vaccine uptake were identified. First, linguistic limitations complicated communication. Local languages lacked precise terminology for MiP; terms used for “placenta” were often euphemistic, and the word for “malaria” (“malungo”) was too general to distinguish between placental, pregnancy-related, or general malaria. Second, participants expressed skepticism about the need for a new vaccine, given the availability of existing preventive methods. Mistrust in biomedical interventions was common, particularly in the absence of visible illness. Concerns about side effects, infertility, and the perception that the vaccine could act as a contraceptive were frequently mentioned, along with a sense of vaccine fatigue. Third, structural and logistical barriers, including long distances to health facilities, cost constraints, and negative experiences with health workers, further discouraged care-seeking and vaccine uptake.

**Prof. Roch Houngnihin** (University d'Abomey-Calavi, UAC, Benin) presented a study conducted in Benin that assessed sociocultural and systemic factors influencing HPV vaccine acceptance. The research was guided by the WHO's Confidence, Complacency, and Convenience model and included interviews and focus groups with parents, adolescent girls, teachers, health professionals, and local leaders in both urban and rural settings. The findings revealed low awareness of cervical cancer and its link to HPV, even among health providers. Vaccine hesitancy was widespread (60% of participants were reluctant to vaccinate), largely due to fears of infertility, side effects, and misinformation, including conspiracy theories about sterilization targeting African, especially Muslim, populations. However, household decision-making dynamics, particularly paternal authority, appeared more influential than religion alone. Additional challenges included a lack of teacher involvement in mobilization, indirect costs despite free vaccines, and weak communication strategies, particularly in remote areas. Social media played a dual role in spreading both accurate information and misinformation.

Discussion points highlighted that vaccine hesitancy is shaped not only by fear of injections but also by the term “vaccine” itself. Moreover, vaccination of adolescent girls remains sensitive due to social concerns and lingering mistrust. Both studies emphasize the importance of context-specific, trusted communication to support the successful roll-out of new vaccines. In rural areas, social media is widely used, but messages must clearly indicate authoritative sources. Nationally, the public prefers health advice from doctors; locally, religious leaders hold influence. Finally, an oral single-dose vaccines would be perceived as more readily accepted. These findings underscore the importance of optimizing delivery platforms and strengthening communication strategies to build public trust in vaccines.

### 5.6 Lessons learned from the HPV vaccine roll-out in sub-Saharan Africa

**Dr. D. Scott LaMontagne** (JSI Research & Training Institute) drew on nearly two decades of global experience with HPV vaccine delivery to highlight key lessons for the future roll-out of MiP vaccines.

Since its introduction, the HPV vaccine has been adopted by 149 WHO Member States and 29 territories [30]. WHO's endorsement of a single-dose schedule [17] improved the feasibility and uptake, particularly in LMICs. However, barriers remain, including vaccine hesitancy and safety concerns in high-income countries, and logistical and financial challenges in LMICs.

Three lessons from the HPV vaccine roll-out inform the MiP vaccine implementation. First, strong stakeholder engagement is essential. Political commitment, involvement of health workers, media, and trusted local voices support vaccine acceptance. Early planning and quality training also correlate with sustained high coverage. Second, communication must be culturally tailored and address age-specific concerns -such as safety, fertility, and international use- while leveraging trusted messengers. Third, successful delivery requires clearly defined target populations, schedules, and strategies (school-based programs to reach adolescents, antenatal care (ANC) visits to reach PW, and outreach for those who are not attending ANC). Given the multiple-dose nature of current MiP vaccines, aligning delivery with other existing health interventions, such as co-administration with HPV vaccine, is key to facilitate implementation. Strong monitoring systems are also needed to track doses and ensure safety.

### 5.7 Lessons learned from intermittent preventive treatment in pregnancy implementation in sub-Saharan Africa

**Clara Menendez** (Barcelona Institute for Global Health, ISGlobal, Spain) presented key learnings from the implementation of IPTp for malaria control in sub-Saharan Africa.

IPTp involves giving antimalarials at scheduled intervals, regardless of infection status. IPTp has been proven efficient, and hence, the WHO currently recommends IPTp-SP to PW in areas with stable malaria transmission, primarily delivered through ANC visits. A randomized trial conducted in Mozambique showed that IPTp-SP reduced clinical malaria during pregnancy by 40% (CI 7.4%–61.2%) [31] and neonatal mortality by 61.3% (95% CI 7.4%–83.8%) [32]. The cost per disability-adjusted life year averted was $1.08, making it a highly cost-saving public health measure [33].

However, IPTp coverage remains low. Although 80–90% of PW attend at least one ANC visit, only 44% received three doses of IPTp in 2023 [1], far below the WHO's 80% target. Barriers to uptake include limited access to ANC, stockouts of SP, and missed opportunities during ANC visits. In response, alternative methods of service delivery, such as the community-based delivery approach, are being implemented to improve coverage. During pregnancy, SP effectiveness can be reduced by concurrent high-dose folic acid supplementation and the emergence of parasite resistance. Interestingly, SP's chemoprevention efficacy may persist despite resistance [34], possibly due to acquired immunity and its antibiotic positive effects on the gut microbiota. Nonetheless, the lack of alternative antimalarials for use in pregnancy remains a growing concern, as described in section 2.

HIV-positive PW face a significantly higher risk of severe malaria, yet current preventive options are limited. SP is contraindicated in this group due to routine cotrimoxazole prophylaxis, which cannot be safely combined with SP. Notably, dihydroartemisinin-piperaquine (DHA-PQ) added to daily cotrimoxazole has been evaluated in this population, demonstrating greater efficacy in reducing parasitemia compared to cotrimoxazole alone [35]. With over one million pregnancies annually in women co-infected with HIV and malaria in sub-Saharan Africa, there is an urgent need for safe and effective prophylactic alternatives.

### 5.8 Accelerated Malaria Vaccine Introduction and Roll-out in Africa (AMVIRA) experience

**Dr. Adidja Amani** (AMVIRA, WHO Regional Office for Africa) presented key insights from the AMVIRA initiative, launched by WHO Regional Office for Africa (AFRO) in January 2024. AMVIRA is a coordination platform supporting Member States in the efficient introduction of malaria vaccines providing tailored technical assistance. Its core strategies include country engagement, stakeholder mapping, and integration of malaria vaccines into routine immunization and antenatal platforms.

AMVIRA reinforces country ownership through the emergency incidence management system that facilitates communication between MoHs, WHO country offices, AFRO and WHO headquarters, ensuring rapid gap identification and course correction. AMVIRA facilitates integrated planning between national malaria control programs and expanded programs on immunization, deploying multidisciplinary experts to support country-specific needs. They also organize regional workshops and learning platforms to enable countries to exchange best practices and troubleshoot implementation challenges. To build trust and harmonize efforts among stakeholders, AMVIRA serves as a convening platform for UNICEF, Gavi, PATH, Clinton Health Access Initiative (CHAI), Africa Centres for Disease Control and Prevention (CDC), President's Malaria Initiative (PMI), John Snow, Inc. (JSI), national non-governmental organizations (NGOs), civil society, traditional and religious leaders, and media partners.

Malaria vaccines RTS,S/AS01 (2021) and R21/Matrix-M (2023) have recently been integrated into routine immunization schedules. To facilitate it, AMVIRA embeds malaria vaccine messaging into ante- and post-natal care counseling, trains staff to identify and refer eligible infants, and incorporates prompts in ANC records to track upcoming infant vaccine needs. These measures increase caregiver awareness, encourage completion of the four-dose schedule, and strengthen the routine immunization “safety net” for newborns. Future plans include tailored ANC communications for maternal malaria vaccines and exploring direct protection for PW, ensuring immunization platforms evolve with emerging recommendations. AMVIRA also contributes to misinformation management via community and religious leaders, combined with health-worker training. Through the WHO malaria vaccine readiness tool and real-time District Health Information Software 2 (DHIS2) dashboards, AMVIRA monitors the current interventions and, through post-introduction evaluations, refines subsequent interventions.

Together, these approaches have accelerated malaria vaccine uptake across the WHO African Region, offering a replicable model for future vaccine introductions in high-burden settings. Dr. Amani highlighted that successful scale-up relies on government commitment, political will, and country financing.

## 6 Panel discussion: Roles and expectations of key stakeholders, and how they can contribute to the successful development and roll-out of MiP vaccines

The panel represented a well-rounded mix of clinical research (**Prof. Akim Adegnika**, FORS, Benin), policy development (**Dr. Adidja Amani,** WHO AFRO), operational implementation (**Scott LaMontagne**, JSI), and civil society and community engagement (**Tendai Mayani**, Mothers2Mothers) representatives. Moderator: **Mary Hamel, M.D.** (WHO).

### 6.1 Increasing the awareness of the need for a MiP vaccine

Participants emphasized the need to position MiP as a public health emergency to generate political will and stakeholder commitment. Unlike HPV, where vaccination is the only viable preventive strategy in settings without screening, MiP already has preventive tools in place, making the introduction of a vaccine more difficult to justify. Clear evidence of added value, feasibility of delivery, and the ability to reach target populations, particularly given challenges with multi-dose schedules, will be essential to secure support. Implementation science and community engagement were identified as key enablers, alongside high-level political buy-in and domestic investment. Over-reliance on donors was seen as a risk to long-term success and ownership. African leadership engagement -both political and technical- was emphasized as a critical factor in overcoming roll-out barriers and ensuring manufacturers remain committed.

### 6.2 Civil society organization engagement for the successful implementation of the MiP vaccine

The role of civil society organizations (CSOs) was discussed as vital for bridging the gap between health systems and communities, especially in remote areas. CSOs can deliver accurate and timely information, helping to communicate policy changes, such as new dosing schedules. Peer-led models, such as mentor mothers in Mothers2Mothers, and trusted community figures, were recommended to reach adolescents and PW.

### 6.3 Real-world evidence data gap

While early-phase trials are progressing, the pharmacovigilance structure is often neglected in Africa. Real-world evidence will be important after a vaccine is proven safe and effective, to assess impact in routine use. This data will be needed to generate early demand, which is critical for community uptake, production planning and financing.

### 6.4 Stakeholders' coordination challenges

Coordination among stakeholders remains a challenge. Manufacturers seek assurances of demand before scaling production, while countries need confirmed supply to commit. WHO and other global actors were called on to mediate this “commitment deadlock.” Early pilot roll-outs and regulatory planning, particularly in regions with known bottlenecks, were encouraged. Cross-country learning and strong country leadership were identified as success factors.

### 6.5 Reduced dosing schedules

Drawing on the HPV experience, participants urged that MiP vaccine trials integrate reduced-dose regimens early on, especially for platforms such as mRNA or DNA vaccines. Phase II non-inferiority trials could evaluate simplified schedules to improve access, cost-efficiency, and implementation feasibility.

## 7 Conclusions and recommendations

Prof. **Ayola Akim Adegnika** (FORS, Benin) and **Flavia D'Alessio** (EVI, Germany) closing remarks highlighted that an optimal MiP vaccine would provide life-long-lasting immunity from a single dose administered to adolescent girls, potentially alongside the HPV vaccine, to facilitate implementation. Such vaccine could close the first trimester protection gap, boost natural immunity, and complement existing malaria interventions.

Several enabling and hindering factors influence the development and introduction of a MiP vaccine. On the enabling side, safe and effective vaccines that prevent malaria in children are being evaluated to prevent MiP with first results awaited in 2026, and promising PM vaccine candidates are in early phases of clinical development. In addition, platforms such as AVAREF and AMVIRA, together with strong political commitment and donor backing could support vaccine roll-out.

**Fig. 1:**
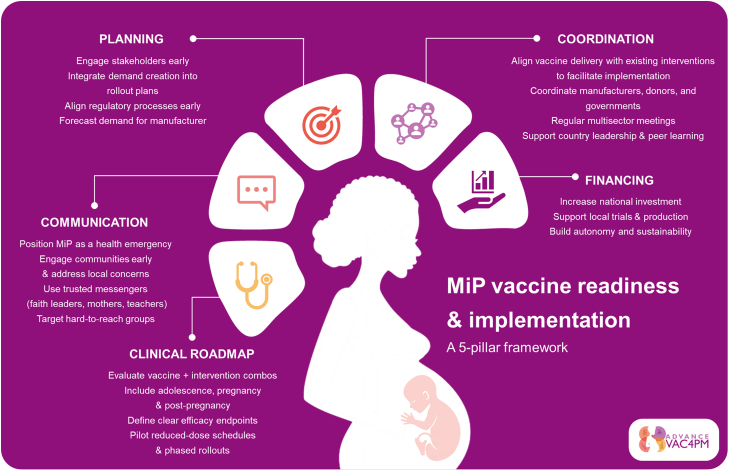
Malaria in pregnancy vaccine readiness and implementation framework based on the key workshop recommendations.

However, several challenges remain: limited awareness of MiP specific impact, scarce real-world evidence, weak pharmacovigilance systems for large-scale deployment, the existence of multiple malaria interventions raising doubts about the added value of a new vaccine, and mistrust in vaccines, especially when targeting adolescent girls. To address these barriers, the authors propose recommendations to support MiP vaccine readiness and implementation (Box 1), summarized in a 5-pillar framework (Fig. 1).

Box 1 **Key recommendations for malaria in pregnancy vaccine implementation.**

**Table:** 

Communication:
• Position MiP as a health emergency to garner comprehensive stakeholder support • Engage communities early and effectively through communication strategies that address local concerns and vaccine safety, reflect sociocultural contexts, and are grounded in community realities. • Develop targeted outreach models for hard-to-reach and underserved populations. • Engage with civil society organizations, including mentor mothers, faith leaders and teachers, to work alongside health care workers as trusted voices. Clinical roadmap: • Early evaluation of vaccine combinations with existing interventions • Include adolescence, pregnancy and post-pregnancy to the critical period to evaluate MiP vaccines. • Incorporate well-defined efficacy endpoints in Phase II trials and reinforce Phase IV clinical research and pharmacovigilance systems to assess safety and real-world effectiveness. • Design clinical trials to explore reduced-dose schedules from the outset, to shorten timelines and improve programmatic feasibility. • Implement phased or pilot introductions of a MiP vaccine to generate early learnings and adapt strategies in real time. Planning • Engage early with all relevant stakeholders. • Integrate demand creation strategies. • Begin regulatory alignment early, particularly in countries where past experiences have shown delays in market authorisation. • Establish mechanisms to estimate demand early, thereby facilitating smoother manufacturer planning and procurement processes. Coordination • Align vaccine delivery with other existing health interventions, such as co-administration with HPV vaccine, to facilitate implementation. • Foster better coordination between manufacturers, donors, procurement agencies, and national governments to resolve supply-demand gridlocks. • Establish regular, structured engagement with multisectoral stakeholders to build a shared vision from the start. • Ensure that countries remain in the lead and promote peer learning and coordinated communication between countries. Financing • Increase national investment from African governments to support clinical trials, vaccine production, and sustainable introduction efforts, thereby promoting national-level responsibility, maintaining leadership engagement, and enhancing local autonomy.

Alt-text: Box 1

## CRediT authorship contribution statement

**Flavia D'Alessio:** Writing – review & editing, Supervision, Conceptualization. **Josiane Honkpehedji:** Writing – review & editing, Conceptualization. **Sodiomon Sirima:** Writing – review & editing. **Benoit Gamain:** Writing – review & editing. **Nielsen Morten:** Writing – review & editing. **Nicaise Ndam:** Writing – review & editing. **Benjamin Mordmüller:** Writing – review & editing. **Alassane Dicko:** Writing – review & editing. **Rhanda Adechina:** Writing – review & editing. **Ange Dossou:** Writing – review & editing. **Jobiba Chinkhumba:** Writing – review & editing. **Mandeep Kaur:** Writing – review & editing. **Roch Houngnihin:** Writing – review & editing. **D. Scott LaMontagne:** Writing – review & editing. **Clara Menendez:** Writing – review & editing. **Adidja Amani:** Writing – review & editing. **Tendai Mayani:** Writing – review & editing. **Mary J. Hamel:** Writing – review & editing. **Elvira Carrió:** Writing – review & editing, Writing – original draft. **Aicha Sayeh:** Writing – review & editing, Conceptualization. **Ayola Akim Adegnika:** Writing – review & editing, Conceptualization.

## Funding

The workshop “Strategies for using malaria vaccines to prevent malaria in pregnancy (MiP)” and this workshop report are produced in the frame of the ADVANCE_VAC4PM project.

This project is funded by the European Union. Views and opinions expressed are however those of the author(s) only and do not necessarily reflect those of the European Union or the European Health and Digital Executive Agency. Neither the European Union nor the granting authority can be held responsible for them.

## Declaration of competing interest

The authors declare the following financial interests/personal relationships which may be considered as potential competing interests: Morten Agertoug Nielsen reports financial support was provided by European Commission. Morten Agertoug Nielsen reports a relationship with AdaptVac that includes: board membership, employment, and equity or stocks. Morten Agertoug Nielsen has patent #SpyTag PA 2015 70019 issued to assignee. Benoit Gamain is the vaccine inventor of the PRIMVAC placental malaria antigen candidate. Flavia D’Alessio, Aicha Sayeh, Ayola Akim Adegnika, Josiane Honkpehedji, Benjamin Mordmüller, Nicaise Ndam, Sodiomon Sirima and Mandeep Kaur receive financial support from the European Union as part of the ADVANCE-VAC4PM project not only for this workshop organisation, but also to further develop the two antigens described in the report (PRIMVAC and PAMVAC-cVLP).

Other authors declare that they have no known competing financial interests or personal relationships that could have appeared to influence the work reported in this paper.

## Data Availability

No data was used for the research described in the article.

## Glossary

ADVANCE_VAC4PM: Advance Vaccine for Placental Malaria project funded by the European Union
ACT: Artemisinin based combination therapy
AL: Artemether lumefantrine
AMVIRA: Accelerated Malaria Vaccine Introduction and Roll out in Africa initiative
ANC: Antenatal care
AS AQ: Artesunate amodiaquine
AS MQ: Artesunate mefloquine
ASPY: Artesunate pyronadine
AVAREF: African Vaccine Regulatory Forum
CDC: Centres for Disease Control and Prevention
CHAI: Clinton Health Access Initiative
CSO: Civil society organizations
CSA: Chondroitin sulfate A
DHIS2: District Health Information Software 2
DHA: Dihydroartemisinin
EPI: Expanded Programme on Immunization
EVI: European Vaccine Initiative
FORS: Fondation pour la Recherche Scientifique Benin
GLA-SE: Glucopyranosyl lipid adjuvant stable emulsion
Gavi: Gavi, the Vaccine Alliance
GRAS: Groupe de Recherche Action en Santé, Burkina Faso
HIV: Human immunodeficiency virus
ICER: Incremental cost effectiveness ratio
INSERM: Institut National de la Santé et de la Recherche Médicale France
IPTp: Intermittent preventive treatment in pregnancy
IPTp SP: Intermittent Preventive Treatment in pregnancy with Sulfadoxine Pyrimethamine
ITN: Insecticide treated bed nets
JSI: John Snow Inc
LMIC: Low and middle income countries
MNTE: Maternal and Neonatal Tetanus Elimination
MiP: Malaria in pregnancy
MoH: Ministry of Health
MRTC: Malaria Research and Training Center
NGO: Non-government organizations
NRA: National Regulatory Authorities
PAMVAC: Pregnancy Associated Malaria Vaccine
PM: Placental malaria
PMI: President's Malaria Initiative
PRIMVAC: VAR2CSA derived placental malaria vaccine candidate
PW: Pregnant women
PSPQ: Programmatic Suitability for Prequalification
R21/Matrix M: Malaria vaccine recommended for pediatric use by WHO since 2023
RUMC: Radboud University Medical Center, The Netherlands
RTS S AS01: Malaria vaccine recommended for pediatric use by WHO since 2021
SHuffle®: Genetically engineered strain of *Escherichia coli* designed for cytoplasmic expression of disulfide bonded proteins
SP: Sulfadoxine Pyrimethamine
PfSPZ: Plasmodium falciparum sporozoite
TCV: Tetanus containing vaccine
UAC: University of Abomey-Calavi, Benin
UNICEF: United Nations International Children's Emergency Fund
VLPs: Capsid virus like particles
WOCBP: Women of childbearing potential
WHO: World Health Organization
